# FrlP, an ABC type I importer component of *Bacillus subtilis*: regulation and impact in bacterial fitness

**DOI:** 10.1128/jb.00320-25

**Published:** 2025-10-30

**Authors:** Inês C. Gonçalves, Ana Pontes, Carla Gonçalves, Isabel de Sá-Nogueira

**Affiliations:** 1 Microbial Genetics Laboratory, UCIBIO, Department of Life Sciences, NOVA School of Science and Technology, Universidade Nova de Lisboa 449390, Caparica, Portugal; 2 Associate Laboratory i4HB, NOVA School of Science and Technology, Universidade Nova de Lisboa 449390, Caparica, Portugal; 3 Yeast Genomics Laboratory, UCIBIO, Department of Life Sciences, NOVA School of Science and Technology, Universidade Nova de Lisboa 449390, Caparica, Portugal; The Ohio State University, Columbus, Ohio, USA

**Keywords:** fructosamines, ABC type I importer, FrlP *B. subtilis*, gene regulation, fructosevaline

## Abstract

*Bacillus subtilis* is able to catabolize fructosamines, also known as Amadori rearrangement products. The *frlBONMD-frlP* operon mediates this process and is subjected to specific and global regulation. Although the degradation pathway favoring α-glycated amino acids is known, the mechanisms of substrate uptake have remained unclear. In this study, mutagenic and functional analyses revealed that FrlONM, a type I ABC importer, along with the nucleotide-binding domain (NBD) FrlP, is required for the uptake of fructosevaline. Transcriptional and translation *frlP-lacZ* fusions indicated that *frlP* is induced by fructosevaline and negatively regulated by the FrlR repressor. In addition, we show that MsmX, a multitask NBD of *B. subtilis*, is also able to serve as an energy motor of this type I ABC importer and that its presence alongside FrlP is vital for optimal growth on fructosevaline. To address the physiological significance of this functional redundancy, we assessed the distribution of ABC type I NBDs FrlP and MsmX across the *Bacillaceae* family. MsmX is homogeneously distributed in the *Bacillaceae* family tree, while FrlP is restricted to the *Bacillus subtilis* group, suggesting that the presence of FrlP together with other components of the fructosamines operon is important for bacterial fitness in plant-associated ecological niches.

**IMPORTANCE:**

*Bacillus subtilis* is widely applied in the industry as a microbial cell factory, as a biofertilizer for sustainable agriculture, in the animal feed industry and as human probiotic. In its natural environment, *B. subtilis* helps to shape the gut microbiome and the phytomicrobiome. Fructosamines, or Amadori rearrangement products, are ubiquitously found in nature and serve as precursors of toxic cell end-products implicated in the pathology of human diseases. This study provides a solid contribution to a deep knowledge of transport mechanisms, genetic regulation, and physiological relevance of fructosamines utilization in *B. subtilis*. Moreover, it highlights an unusual strategy to adapt to alterations in nutrient availability by swapping the energy providing domain of ABC transporters.

## INTRODUCTION

Ubiquitous across all kingdoms of life, ATP-binding cassette (ABC) transporters share a similar structural architecture which consists of two transmembrane domains (TMDs), that assemble the translocation pore enclosed in the lipid bilayer, and two nucleotide binding domains (NBDs or ATPases) that energize the transport system via ATP hydrolysis (1–4). Contrary to TMDs, NBDs share sequence and folding conservation, especially motifs responsible for accommodation and coupling of the ATPase activity (4). ABC exporters are present across all domains of life, while importers are exclusive to prokaryotes and plants (5). ABC Type I importers are accountable for the transport of primary metabolites needed in high quantities, such as sugars and amino acids, and they require a third component, a solute-binding protein (SBP), which in Gram-negative bacteria is located in the periplasmic space while in Gram-positive organisms is anchored to the plasma membrane through a N-terminal extension (4, 6). In bacterial genomes, all elements of ABC transporters are generally encoded in the same DNA region or clustered together in an operon (7, 8). That is the case of the prototype of bacterial ABC Type I importers the MalEFGK_2_ from *Escherichia coli* responsible for maltose uptake (8, 9).

**Fig 1:**
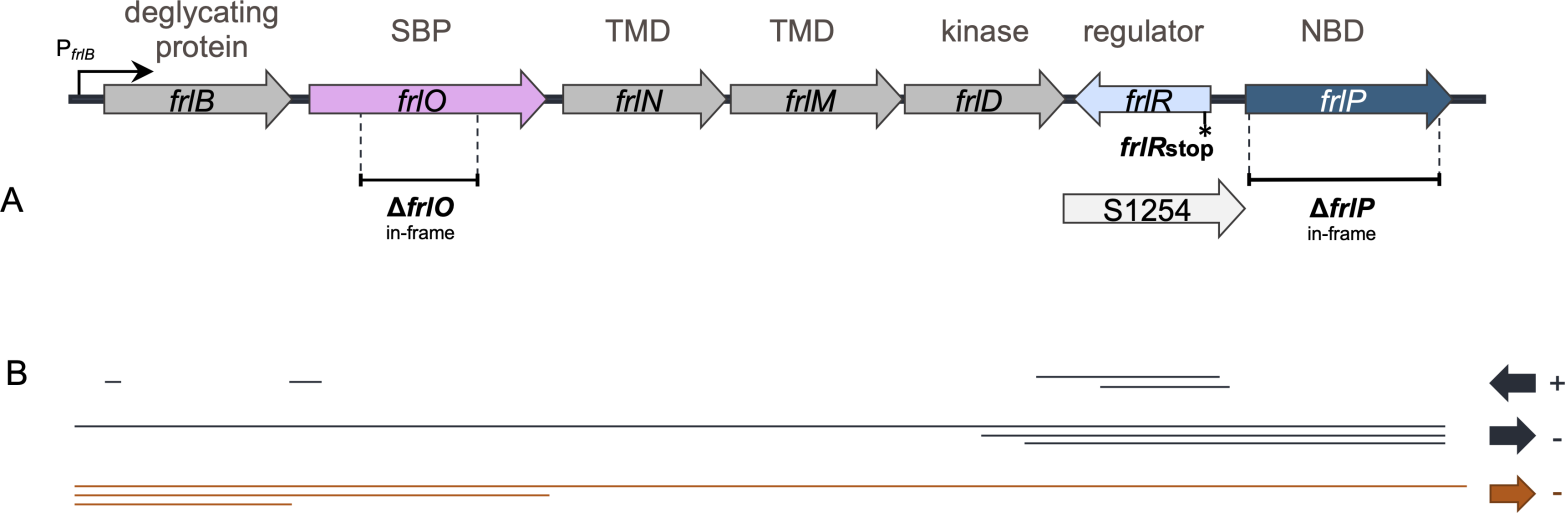
Representation of the *frlBONMD* operon genomic region enclosing genes coding for the glycosidase FrlB, the substrate binding protein (SBP) FrlO, permeases (TMDs) FrlN and FrlM, the kinase FrlD, the repressor of the operon, FrlR, and the nucleotide binding domain (NBD) FrlP. The putative antisense RNA S1254 is located between *frlD* and *frlP* sequences. The promoter of the operon P*_frlB_* is indicated by an arrow. (**A**) Schematic representation of *frl* operon mutations constructed in this work: in-frame deletion Δ*frlO*; nonsense point mutation *frlRstop*; and in-frame deletion Δ*frlP*. (**B**) Transcription units identified by ChIP-chip experiments (19), retrieved from SubtiWiki (http://www.subtiwiki.uni-goettingen.de), are represented below as solid dark lines and arrows point to the direction of transcription. Solid orange lines indicate mRNA molecules detected by northern blot analysis using a *frlB* probe after cell growth in minimal medium with glucose (20).

The chromosome of the well-studied Gram-positive bacterium *Bacillus subtilis* revealed exceptions displaying several independent but incomplete ABC carbohydrate transport systems lacking the NBD and two orphan NBDs, MsmX and FrlP (formerly YurJ) (7). Studies have shown that the orphan ATPase MsmX is able to energize six different carbohydrate uptake systems, namely, MdxEFG (10), AraNPQ (11), GanSPQ (12), RhiLFG and YtcPQ-YteP (12, 13), and MelECD (14), involved in the uptake of maltodextrins, arabino-oligosaccharides, galacto-oligosaccharides, galacturonic acid oligomers and/or rhamnose-galacturonic acid disaccharides, and melibiose, respectively. Recently, the use of AlphaFold to predict ABC transport systems in *B. subtilis* confirmed the assembly of these complexes and identified an additional putative LplABC–MsmX assembly (15). Due to the extended capacity of interaction and energization of a large spectra of transporters, MsmX was denominated as multitask or multipurpose (11). The less studied FrlP ATPase is encoded in the downstream region of an operon responsible for fructosamine utilization (Fig. 1) (16, 17). This operon comprises *frlB*, encoding a deglycating protein, *frlD*, encoding a kinase, putative transporter genes (*frlONM*), and a convergently encoded repressor gene, *frlR*, co-localized with a non-coding RNA, S1254 (16, 17). Recent computational modeling with AphaFold-Multimer has provided structural predictions for the FrlONM transporter complex (18).

Fructosamines, or Amadori products, constitute the first stable intermediates of the Maillard reaction, being spontaneously produced when the carbonyl group of reducing sugars non-enzymatically reacts to the free amine group of amino acids or proteins (21). Amadori products are ubiquitously found in nature, such as in humic substrates, fruits and vegetables, and several stored or processed foods (22, 23). In certain ecological niches, for example, the human and animal gut or the rhizosphere, Amadori products shape the microbiome (22, 24, 25). *E. coli*, yeasts, and mammals are subjected to intracellular protein glycation, so it is plausible that the same could happen in *B. subtilis*, driven by the presence of glucose 6-phosphate(P), resulting from the metabolism of other carbon sources (16). Microorganisms can metabolize fructosamines through deglycation by two types of enzymes: amadoriases (oxidases) or fructosamine kinases, the latter being the mechanism observed in both *E. coli* and *B. subtilis* (16, 17, 26). Fructosamine kinases phosphorylate the Amadori product at C-6 and the deglycase enzyme catalyzes the breakdown of the fructosamine 6P into, e.g., glucose 6P and a free amine (16, 26, 27). Furthermore, it has been shown that both organisms are able to sustain growth on Amadori products (17, 26). However, substrate specificity differs between them; unlike *E. coli*, *B. subtilis* kinase FrlD and deglycase FrlB have substrate specificity toward α-glycated amino acids instead of ε-glycated lysine (27).

Although some efforts have been made to understand the physiological significance of the *frlBONMD* gene products in *B. subtilis* (17, 27), targeted gene studies are still lacking, especially regarding *frlP* and its involvement in this fructosamine utilization pathway. Our previous studies have shown that FrlP is able to energize AraNPQ and GanSPQ in the import of arabinan and galactan derived products, respectively, upon ectopic expression controlled by a synthetic promoter (12, 28). Here, we report, by genetic and functional studies, the involvement of FrlONM-FrlP in the uptake of fructosevaline. Moreover, the involvement of MsmX in this sugar amines utilization pathway is revealed by genetic analysis. Transcriptional and translational studies of *frlP* expression in the presence of different substrates shed light on the putative exchangeability between FrlP and MsmX and their roles in bacterium fitness. The importance of FrlP presence in the cell was further investigated by exploring the phylogenetic distribution of both FrlP and MsmX among the *Bacillaceae* family.

## RESULTS

### FrlONM are involved in the uptake of fructosevaline

**Fig 2:**
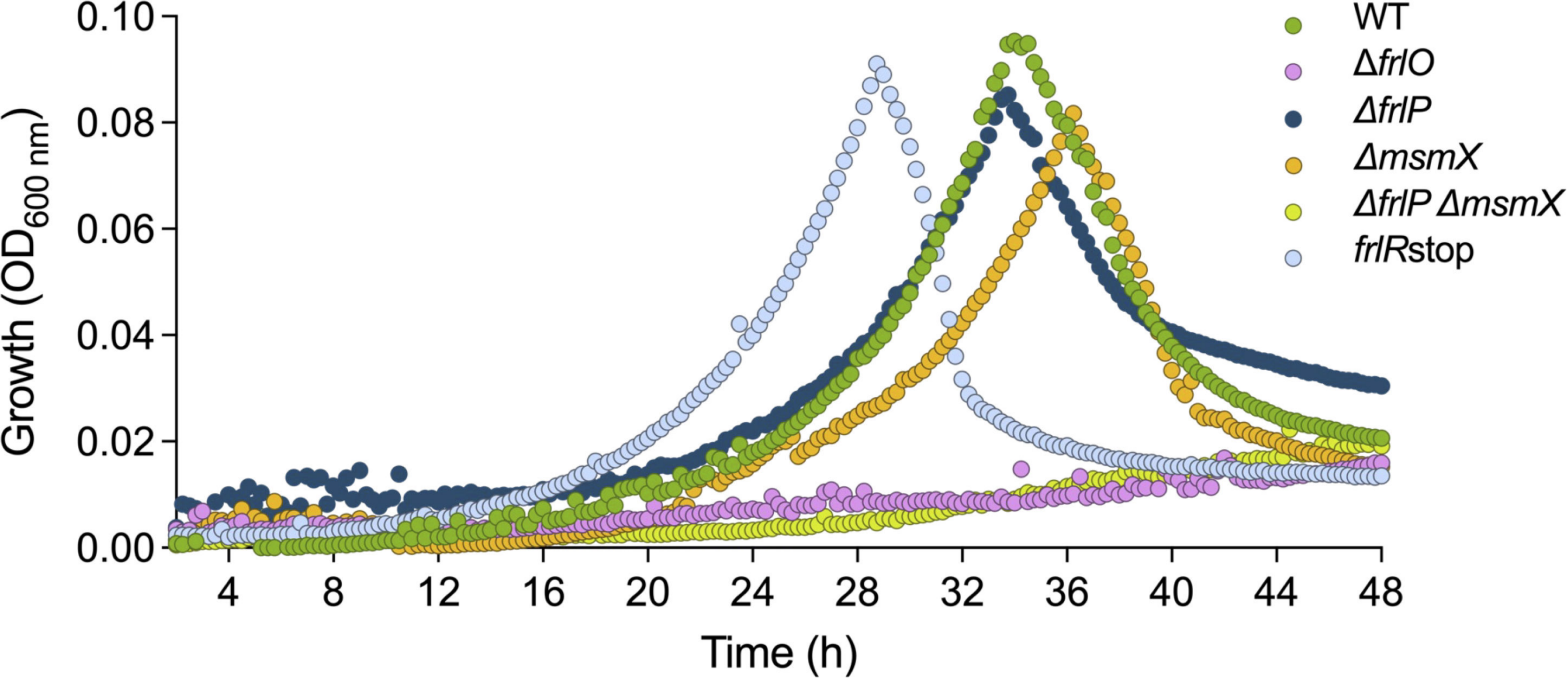
Growth curves of *B. subtilis* WT (dark green), *frlRstop* (light blue), Δ*frlO* (pink), Δ*msmX* (orange), Δ*frlP* (dark blue), and Δ*msmX*Δ*frlP* (light green) strains in M9 minimal medium supplemented with 22 µg mL^−1^ CAF and 2 mM fructosevaline. Shown is one representative growth curve of each strain. Two independent growth experiments were performed using technical triplicates, except for the Δ*frlP*, for which four independent experiments were conducted. All growth curves are shown in Fig. S1C.

The *frlBONMD* operon (Fig. 1) has previously been reported as necessary for the uptake and utilization of fructosamines based on studies using a *frlBONMD* knockout mutant (16). FrlP has been proposed as the ATPase that couples the transport of fructosamines (15, 29, 30) even though its functional role has not been experimentally validated. Similarly, FrlO has been annotated as the SBP responsible for Amadori delivery to TMDs FrlN and FrlM (15, 18). However, previous studies used a raw mixture of the Amadori product fructosyl-arginine that retained residual glucose and arginine (17) or small amounts of product obtained by an undisclosed protocol (30). Since *B. subtilis* kinase FrlD and deglycase FrlB have substrate specificity toward α-glycated amino acids (27), in this study, we used fructosevaline (mixture of diastereomers; CymitQuimica). To ascertain the role of FrlONM in the import of fructosamines, a markerless in-frame *frlO* deletion mutant was constructed to prevent polar effects on the transcription of the operon downstream genes (Fig. 1A). The ability of the mutant strain to sustain growth with fructosevaline as sole carbon and nitrogen source was determined and compared to the WT strain (Fig. 2). The results showed that Δ*frlO* mutant failed to grow on fructosevaline, confirming that FrlO is essential for the delivery of this Amadori product to the TMD domains of the FrlONM transporter complex.

### FrlP and MsmX are two NBDs that energize the FrlONM importer

The *frlP* gene encoding a putative NBD domain of the FrlONM was previously shown to be part of the *frlBONMD* operon by tiling arrays and northern blot (19, 20; Fig. 1B). To determine if FrlP is responsible for energizing the FrlONM transport system, a deletion mutation Δ*frlP* was constructed and the resulting strain was assessed for its ability to grow on fructosevaline as the sole carbon and energy source (Fig. 2). The deletion did not impair the ability of the mutant Δ*frlP* cells to grow on fructosevaline, suggesting that other NBD(s) might energize the FrlONM transport system. Previously, our laboratory showed that ectopic expression of *frlP*, under the control of a synthetic promoter P_spank(hy)_, complements the absence of the multitask NBD MsmX in the oligosaccharides transport systems AraNPQ and GanSPQ (28). Thus, we tested the ability of an *msmX* null mutant (Δ*msmX*) to grow on fructosevaline as the sole carbon and energy source. Remarkably, the impact of the *msmX* deletion on bacterial growth was very similar to that displayed by the *frlP* deletion (Fig. 2). We, therefore, generated a *msmX* and *frlP* double mutant which showed to be unable to grow on fructosevaline (Fig. 2). These results strongly suggest that neither FrlP nor MsmX function as the exclusive energy generator components of the fructosamines transporter complex. Under the conditions tested, both NBDs (FrlP and MsmX) are functionally interchangeable and capable of energizing the FrlONM transporter.

**Fig 3:**
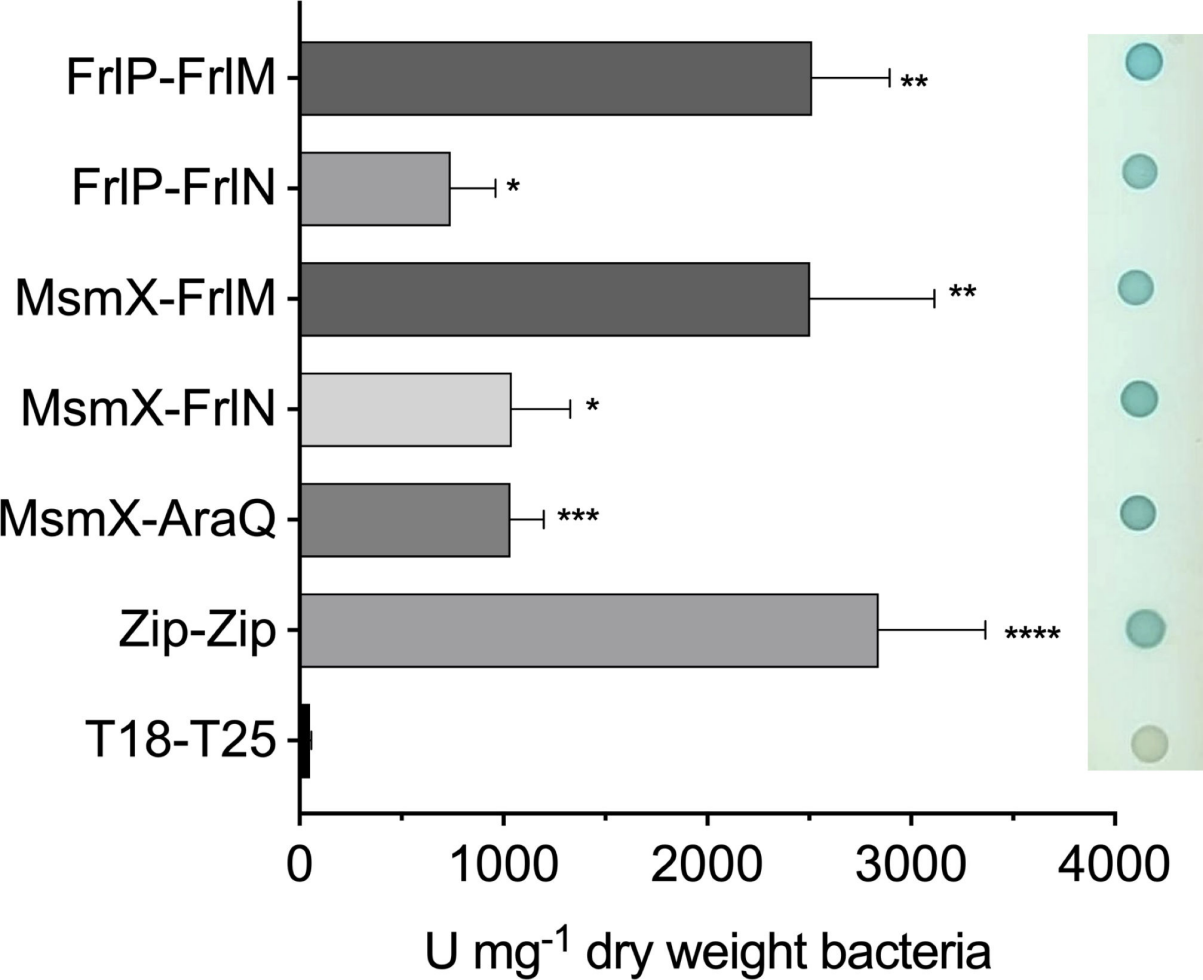
Quantitative and qualitative analysis of protein-protein interaction by B2H assays. Interaction between T18 and T25 fragments expressed from empty pUT18 and pKT25 vectors was used as negative control. Zip-Zip and MsmX-AraQ interactions were used as positive controls. At least three independent experiments are represented for each interaction; the error bars represent the standard deviation of the mean. β-Galactosidase activity was calculated as described in Material and Methods, and it is represented in U mg^−1^ dry weight bacteria. Statistical significance between β-galactosidase activity of the different interactions and the negative control is indicated (*P < 0.05; **P < 0.01; ***P = 0.001; ****P < 0.0001). The right panel shows qualitative results from the respective spotted *E. coli* cultures.

To confirm this line of evidence, protein-protein interactions of MsmX and FrlP with TMDs FrlN and FrlM were probed by a bacterial two-hybrid system (B2H) in *Escherichia coli* (31). For validation, the interaction between MsmX and AraQ, a TMD from the AraNPQ importer, was also tested. The results revealed that both FrlP and MsmX can establish *in vivo* contacts with TMDs FrlN and FrlM, corroborating their functional interchangeability as NBD domain of the importer FrlONM (Fig. 3).

### Expression of *frlP* is induced by fructosevaline and negatively regulated by FrlR

Repressors CodY and FrlR were previously shown to bind directly to the *frlB* promoter region, controlling the expression of the fructosamine operon (17, 32, 33). More recent studies contradict these observations proposing that FrlR functions as an activator (30). To clarify the controversy and analyze the regulation of *frlP,* we constructed a nonsense mutation at the 5′-end of the gene (*frlRstop*; Fig. 1A). When growing this strain in the presence of fructosevaline, the mutant reached its peak of growth earlier than the WT strain (Fig. 2; Fig. S1C) suggesting that FrlR acts as a repressor. This behavior is identical to that observed with the repressor of the ε-fructoselysine operon of *E. coli* (34).

**Fig 4:**
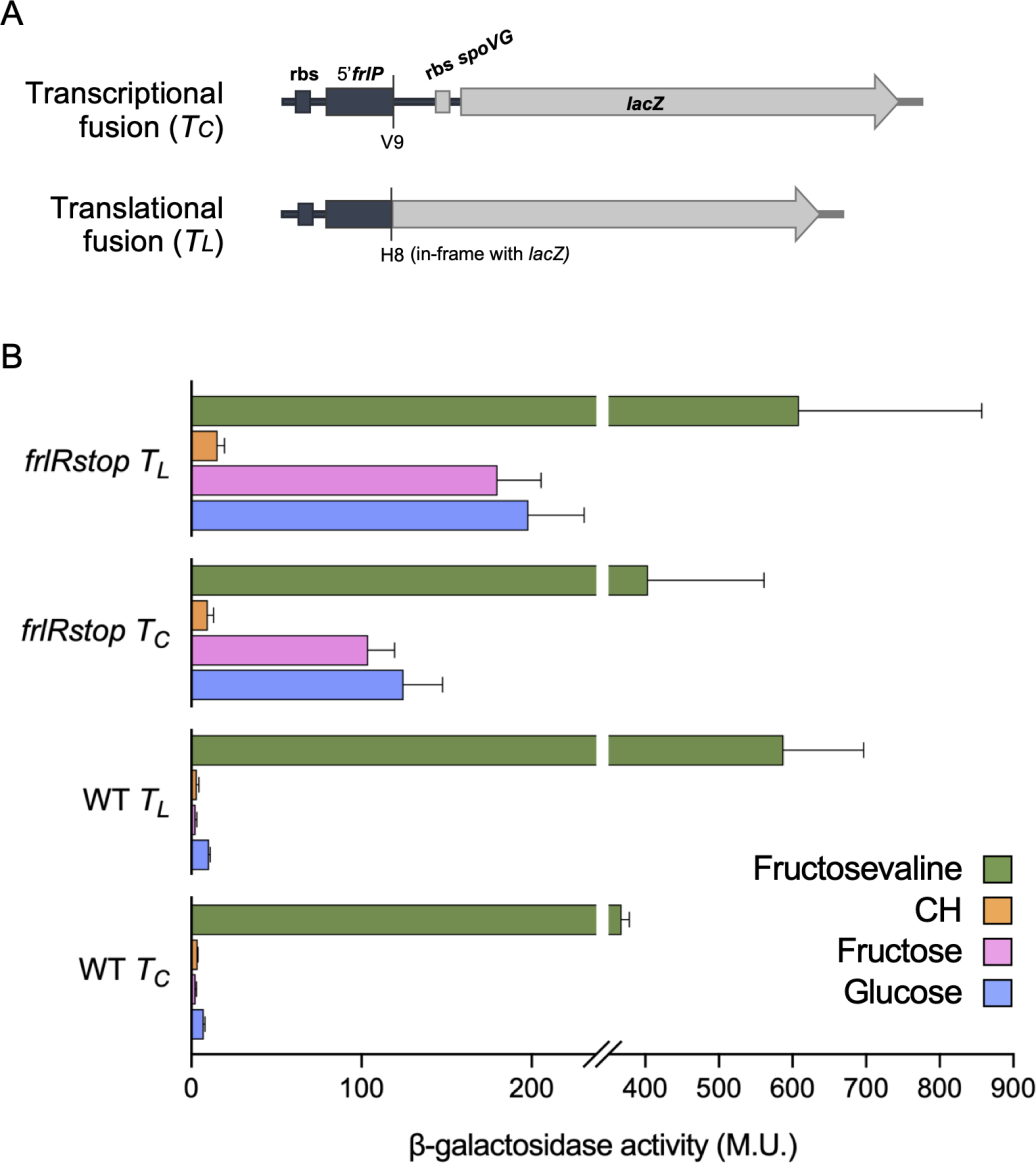
Transcriptional (T*_C_*) and translational (T*_L_*) gene fusions constructed for measurements of *frlP* expression. (**A**) Genomic representation of transcriptional and translational fusions of the 5′-end region of *frlP* gene to the *lacZ* gene from *E. coli*. (**B**) Quantification of *frlP* expression in the WT and *frlRstop* genetic backgrounds. β-Galactosidase activity of *frlP-lacZ* strains grown in M9 minimal medium supplemented with 22 µg mL^−1^ CAF and 1 mM glucose, 1 mM fructose, 1% (wt/vol) casamino acids (CH), or 2 mM fructosevaline was measured in cells recovered at approximate growth peaks (Fig. S2). M. U.—Miller Units. At least two biological replicates are represented. In glucose, β-galactosidase activity of T*_L_* fusion in the WT background (strain ISN129) was measured in two independent experiments, while expression data from the remaining strains include at least three independent experiments. Assays in fructose and CH were measured three and two times, respectively, for all strains. β-Galactosidase activity in fructosevaline was repeated two times, except for the WT strain, for which three independent experiments are included.

Expression of *frlP* is driven by the promoter P*_frlB_* (19, 20; Fig. 1A); however, by bioinformatic analyses, a putative promoter was found upstream from the *frlP* coding region (19). In addition, although FrlP can functionally substitute MsmX in the import of arabinotriose when ectopically expressed, the expression in its own *locus* does not lead to protein accumulation in the presence of glucose, arabinose, or arabinotriose as sole carbon and energy source (12). To answer these questions and understand how *frlP* is expressed in its own locus, transcriptional and translational fusions of the *frlP* 5′-end to the *lacZ* gene of *E. coli* were constructed (Fig. 4A). The fusions were analyzed in both WT and *frlRstop* nonsense mutant backgrounds grown in the presence of different carbon sources: fructosevaline, glucose, fructose, and casaminoacids (Fig. 4B). β-Galactosidase activity assays showed very low level of *frlP* expression in the WT background when grown in glucose, fructose, or casamino acids, both at transcriptional (T*_C_*) and translational (T*_L_*) levels, whereas a high increase of about 150-fold (T*_C_*) and 225-fold (T*_L_*) in the expression was observed in the presence of fructosevaline when compared to fructose. This result suggests that fructosevaline, or one of its metabolic products, is a strong inducer of *frlP* expression. In the depleted FrlR mutant (*frlRstop*), *frlP* expression increased about 18-fold (T*_C_* and T*_L_*) in the presence of glucose and 40 (T*_C_*) to 70-fold (T*_L_*) in the presence of fructose, further indicating that FrlR acts as a repressor, impairing transcription and translation in the presence of these sugars. In the presence of casamino acids, repression is only partially relieved, which is consistent with the role of CodY, as a negative regulator of the fructosamines operon (17, 32, 33). In sum, our data correlate with previous studies conducted with the promoter P*_frlB_* (17), indicating that *frlP* expression is induced by fructosevaline and negatively regulated by FrlR and CodY.

### FrlP is restricted to the *Bacillus subtilis* group

**Fig 5:**
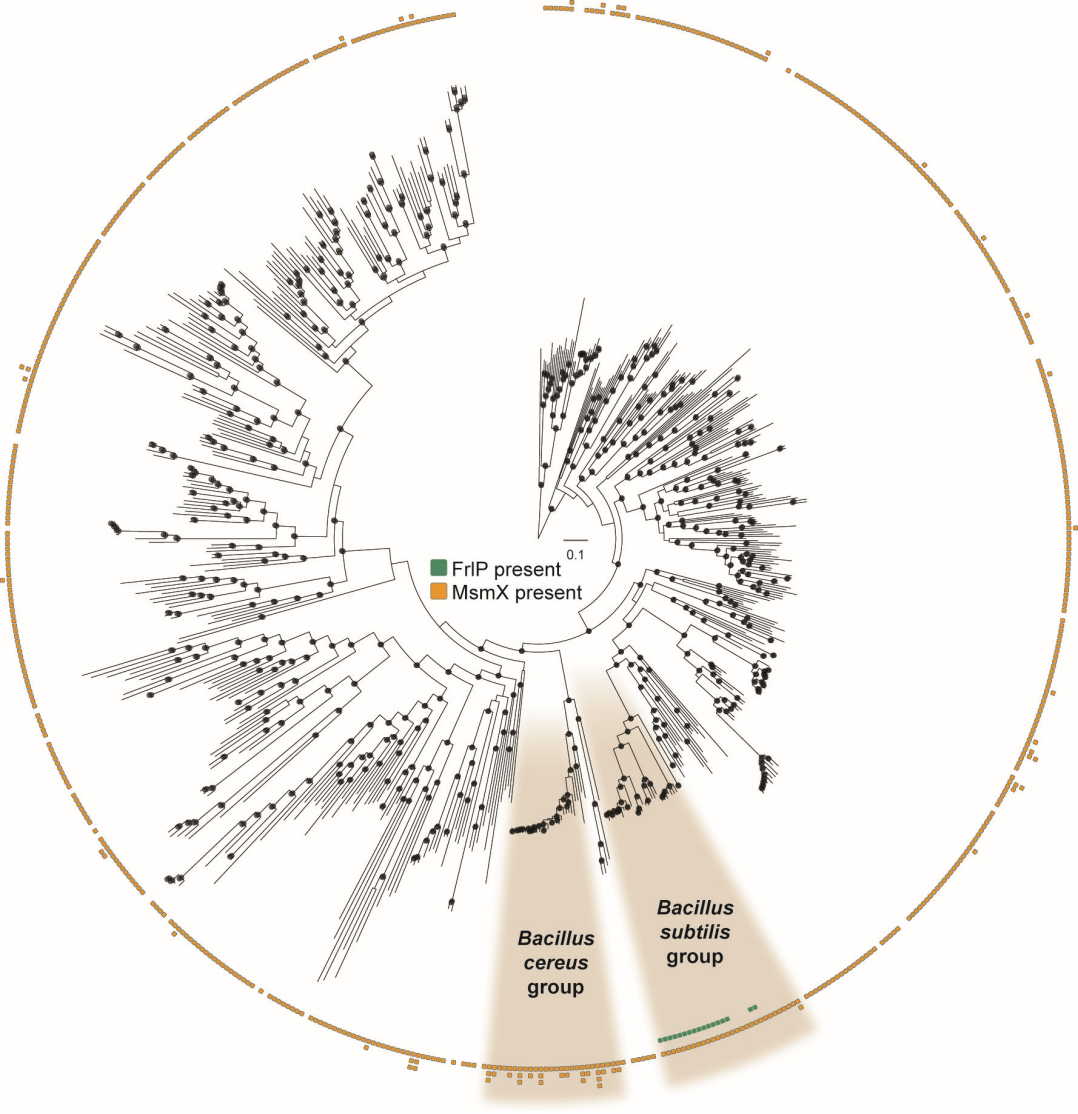
Maximum likelihood phylogenomic tree comprising 629 species from the *Bacillaceae* family inferred from the concatenated alignment of 100 single-copy orthogroups and rooted with *Staphylococcus aureus*. The species from the *Bacillus subtilis* and *Bacillus cereus* groups are highlighted. Presence/absence of *msmX*/*frlP* and number of protein-coding genes are depicted in the ring area.

To elucidate the distribution of ABC type I NBDs FrlP and MsmX proteins amongst the *Bacillaceae* family, we inferred a phylogenomic tree (Fig. 5; Fig. S3; Table S1) which recapitulates the phylogenetic relationship among the main lineages within *Bacillaceae* (35).

**Fig 6:**
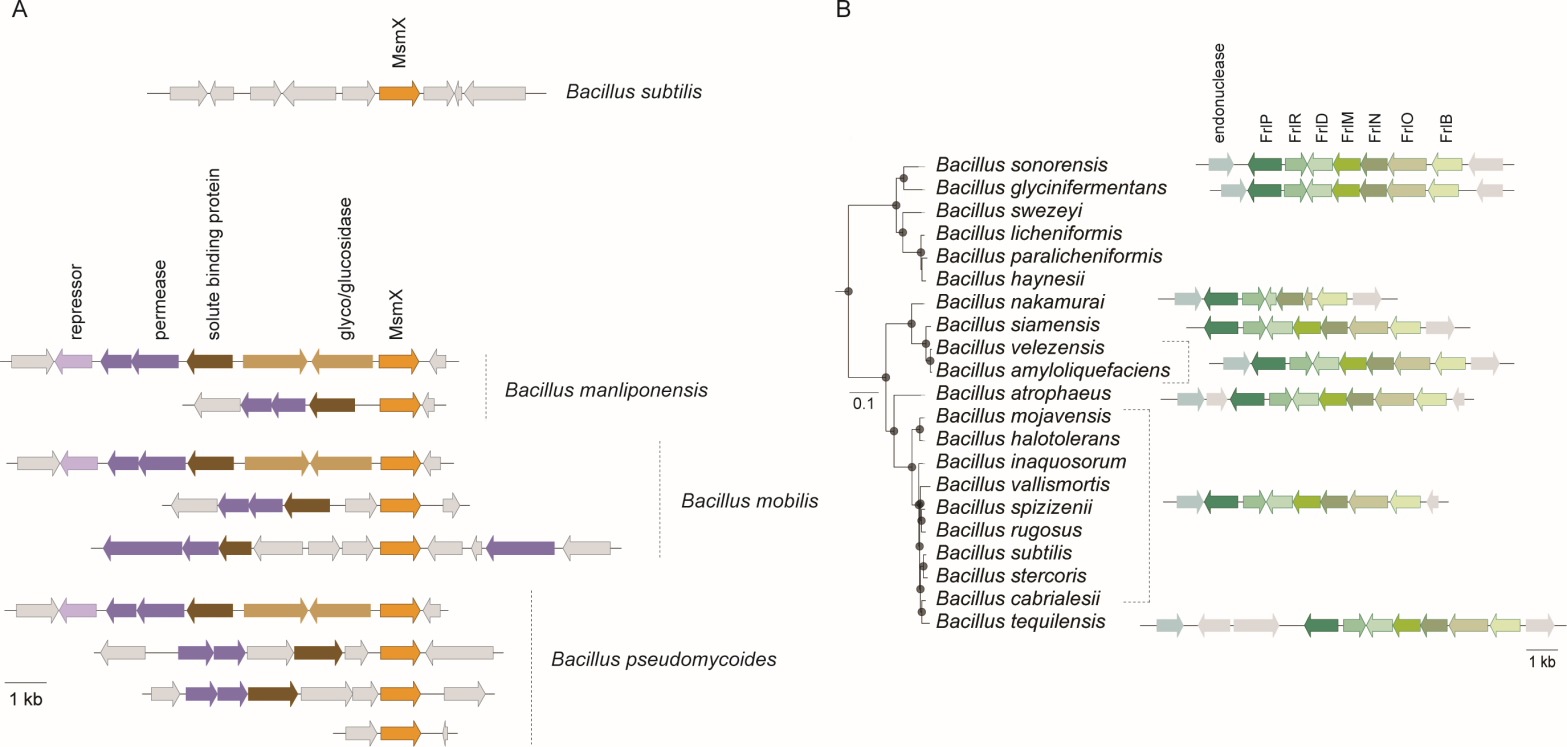
Genomic context of *msmX* and *frlP* genes. (**A**) Synteny of the *msmX* gene in *B. subtilis* and in three species of the *Bacillus cereus* group that contain more than one copy of the gene: *B. manliponensis*, *B. mobilis,* and *B. pseudomycoides*. Gene products are designated as annotated in the respective genomes. Genes sharing the same color encode proteins with similar predicted functions; gray arrows represent genes that are not related to sugar transport and, therefore, are not relevant for this study. (**B**) Pruned tree of the *Bacillus subtilis* group and respective genomic organization of the *frlP* cluster for each species. Orthologous genes are represented by the same color.

We further assessed the distribution of the two ABC type I NBDs (FrlP and MsmX) among the *Bacillaceae* family using a sequence homology-based strategy (36) and found that *Bacillus subtilis* and *Bacillus cereus* groups exhibit the greatest variation in the distribution patterns of these proteins. We noted that MsmX is virtually present in almost all species analyzed and that the *B. cereus* group is particularly enriched in MsmX paralogs (Fig. 5). The representation of *msmX* synteny in three different *B. cereus* group species (Fig. 6A) shows that *msmX* is located next to or in the vicinity of genes that code for components of an ABC importer, contrary to *B. subtilis* 168, in which *msmX* is isolated in the genome as an orphan ATPase (7, 11). On the other hand, FrlP is strictly present in the *B. subtilis* group, in 17 different species (Fig. 5), and its distribution in the phylogenetic tree suggests that an operon loss occurred in the most recent common ancestor of *B. swezey*, *B. licheniformis*, *B. paralicheniformis,* and *B. haynesii* (Fig. 6B). The *frlBONMD-frlR-frlP* cluster synteny is conserved in all species, except in *B. nakamurai* in which gene loss and pseudogenization appears to be occurring (Fig. 6B). Furthermore, all organisms that contain FrlP also contain MsmX (Fig. 5), suggesting the importance of the latter to *B. subtilis* fitness. Species with MsmX duplications were isolated from diverse environments (substrate of isolation of all genome entries on NCBI, BioSamples, as of May–September 2024), indicating a wide ecological distribution (Table S2). Members of the *B. subtilis* group containing FrlP were frequently found in soil and plant-associated habitats, suggesting that FrlP may be or may have been important for Amadori utilization, especially in plant-related surroundings.

## DISCUSSION

In the present study, the physiological importance of the FrlP ABC type I NBD (ATPase) was investigated. Information concerning FrlP has only been presumed through its genomic context. Hereby, by generating specific mutations, we show that the FrlONM importer is required to utilize fructosevaline (Fig. 2). Moreover, not only FrlP is able to energize the FrlONM transporter, but so is MsmX, and both NBDs are functionally exchangeable in the presence of fructosevaline (Fig. 2). These observations are supported by *in vivo* protein-protein interaction assays in *E. coli* confirming that both ATPases had affinity toward TMDs FrlN and FrlM and that the interaction was stronger with the latter (Fig. 3). This unexpected finding extends the role of MsmX as a major multitask ABC type I ATPase responsible for carbohydrate import in *B. subtilis* (10–15). Previous reports have shown that *frlP* is expressed in a long transcript that includes *frlBONMD-frlP* driven by the promoter P*_frlB_* (17, 20, 32). However, two other shorter transcription units were also identified (19) (Fig. 1). Contradictory statements concerning the specific regulation of the Amadori operon by the transcription factor FrlR in *B. subtilis* are found in the literature (17, 30). Thus, the construction of a nonfunctional FrlR mutant was imperative to clarify this subject and study the expression of *frlP*. Two lines of evidence indicate that this protein acts as a repressor: (i) the *frlR* nonsense mutation retards the *lag* phase of cells growing in fructosevaline relative to the WT (Fig. 2) and (ii) the same mutation increases expression of *frlP-lacZ* fusions constructed in its own locus when compared to the WT (Fig. 4). The extended *lag* phase in the WT might be due to a presumably delay in Amadori metabolization, as observed for the *E. coli* FrlR homolog mutant (34). In the absence of fructosevaline, the mutant was highly derepressed both at transcriptional and translational levels compared to the WT. The availability of fructosamines most likely decreases the affinity of FrlR to the DNA leading to the derepression of the operon and the consequent expression of *frlP*. The mechanism by which FrlR from *E. coli* is derepressed was proposed to be like that of NagR (YvoA) from *B. subtilis*, a GntR-type transcription factor that negatively regulates genes from the N-acetylglucosamine (GlcNAc)-degrading pathway (34, 37). Phosphorylated effectors GlcNAcP or glucosamine-6-phosphate (GlcNP) allosterically bind to the C-terminal region of NagR, weakening the DNA binding ability of the winged helix-turn-helix (wHTH) DNA-binding domain in the N-terminal region and, therefore, abolishing the repression that was being exerted (37, 38). Graf von Armansperg and colleagues proposed that fructoselysine-6P is the cognate substrate of FrlR in *E. coli* (34). Given the high similarity between the FrlR from *B. subtilis* and the homolog in *E. coli*, including the conservation of some phosphate binding residues (34), FrlR from *B. subtilis* may recognize phosphorylated Amadori compounds as well. This would cause the abolishment of its DNA-binding ability and, thus, allowing the expression of downstream genes necessary for Amadori catabolism, including *frlP*. Deppe et al. (17) showed that Amadori composed by the condensation of glucose and arginine could support growth in *B. subtilis*. Here, we found that *B. subtilis* can grow in the presence of another type of Amadori product, fructosevaline. This observation supports the findings of Wiame et al. (27) who analyzed the *in vitro* properties of the *B. subtilis* FrlB and FrlD enzymes and demonstrated their role in the metabolism of fructosamines linked to the α-amino group of amino acids pointing to the capacity of metabolize a number of diﬀerent substrates. Thus, we propose that the correct name for the *frlBONMD-frlP* operon in *B. subtilis* is fructosamines (or Amadori [17]) operon, instead of fructoselysine operon (30).

**Fig 7:**
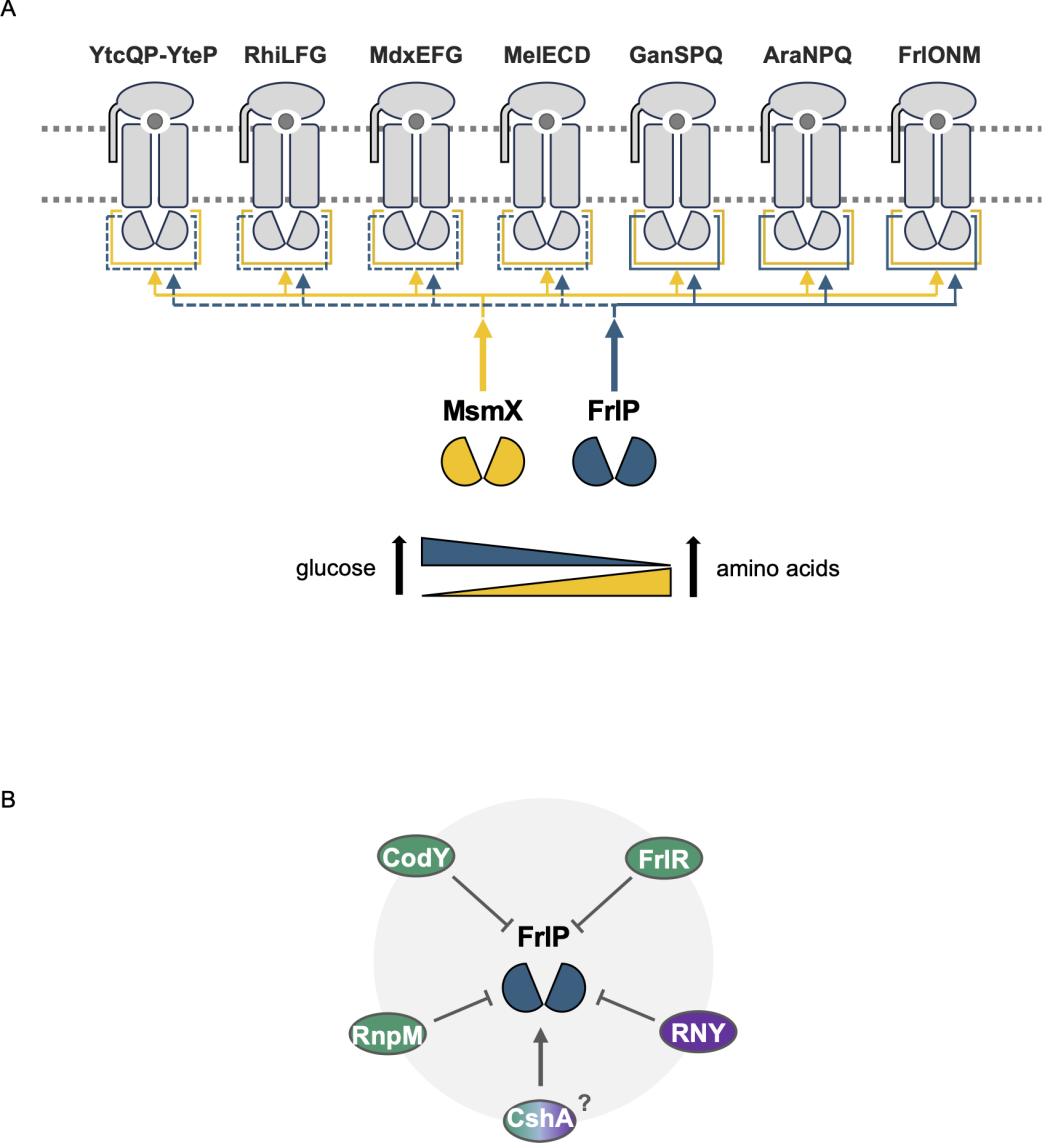
Model for the regulation of FrlP and its impact in the physiology of *B. subtilis*. (**A**) Interplay between NBDs MsmX and FrlP and sugar ABC type I importers in *B. subtilis*. Full lines represent experimentally verified functional energization of the respective transporters; dashed lines represent putative binding of the NBD to the respective transporters. Below is depicted the intracellular concentration of MsmX and FrlP is in response to nutrient availability regulated by global transcription factors CcpA and CodY. In the presence of glucose (left) as carbon and energy source intracellular accumulation of MsmX (depicted by a yellow triangle showing increasing concentration from left to right) is low due catabolite repression of *msmX* exerted by CcpA at the transcriptional level. On the other hand, in response to amino acids (right) as source of carbon and energy, the level of FrlP (depicted by a blue triangle pointing decreasing concentration from left to right) is low due to the action of CodY which transcriptionally downregulates *frlP* expression. (**B**) Regulators directly or indirectly involved in *frlP* expression at transcriptional (green) and posttranscriptional (purple) levels. See discussion for detailed information concerning each player.

Several functional studies have established MsmX as a multitask NBD since it is the energy generator component of several ABC type I sugar importers (10–15). In this study, we show that both MsmX and FrlP participate as NBD of the fructosamines importer FrlONM (Fig. 7). Besides the specific regulation by the FrlR repressor in response to fructosamines and the repression of the general transcription factor CodY triggered by branched-chain amino acids, both exerted at the transcriptional level, other factors, such as RnpM (formerly YlxR) (nucleoid-associated protein modulator of RNA polymerase and RNaseP modulator), RNaseY (essential 5′-end sensitive endoribonuclease), and CshA (*B. subtilis* major RNA helicase), are known to directly or indirectly participate in the regulation of the *frlBONMD-frlP* operon (16, 30, 32, 33, 39, 40). The fructosamines operon is subject to the severe RnpM-dependent transcription repression (41). RNaseY likely degrades *frlBONMD-frlP* mRNA since its depletion leads to an increase in expression of the operon (40), and depletion of CshA severely decreases *frlBONMD-frlP* expression at the mRNA level (20). Similar to that postulated by Deppe and colleagues for the fructosamines operon (17), and based on the results obtained in this study (Fig. 4), we propose that during growth on glucose (or fructose), intracellular glucose 6-phosphate leads to the glycation of proteins and amino acids, fructosamine 6-phosphates inactivates FrlR, which consequently increases expression of FrlP. During growth on casaminoacids, expression of FrlP is decreased via CodY-dependent transcription repression. Conversely, during growth on glucose expression of *msmX* is subjected to repression under the control of global transcriptional repressor CcpA (42). During growth on casaminoacids *msmX* expression is increased (43). This ensures that a functional NBD MsmX or FrlP is present in the cell to energize the fructosamines importer FrlONM (Fig. 7). Since FrlP is also able to substitute MsmX as energy motor for the AraNPQ and GanSPQ, it would be interesting to investigate if the same is valid to the remaining ABC type I importers energized by MsmX (Fig. 7). Remarkably, MsmX and FrlP display a structural C-terminal domain fold like the one present in MalK from *E. coli* that recruits regulatory proteins (15), suggesting additional levels of regulation targeting the two NBDs. Noteworthy, both NBDs FrlP and MsmX are encoded in operons belonging to a class of metabolic operons that are heterogeneously expressed across a clonal population of isogenic siblings (30, 44). Bacterial populations engage in bet-hedging strategies to maximize survival since heterogeneity increases fitness when compared with homogeneous populations (45, 46). The differential expression of FrlP and MsmX in subsets of cells may allow the concomitant import of different carbon sources by the total population (Fig. 7).

*Bacillus* spp., including *B. subtilis*, represent the major genus of the Gram-positive bacterial populations in the soil and rhizosphere, and one of the most widely spread endophytic bacteria (47). Additionally, evidence indicates that *B. subtilis* is part of the normal gut microbiota of animals including humans (48, 49). Although research toward understanding Amadori utilization in bacteria is scarce, the catabolic machinery is complex and involves a different set of enzymes and is present in several organisms found in soils, rhizosphere, and associated with the gastrointestinal tract (22, 50–55). Examples include the capacity to utilize Amadori deoxyfructosyl glutamine, which is dispersed through bacteria of the family *Rhizobiaceae* commonly found in rhizospheres and rotting plant materials (22, 50) or fructoselysine degraded by human intestinal microbiota (51–53). To assess the importance of the presence of FrlP in the cell, given the promiscuous nature of MsmX binding, the phylogenetic distribution of both FrlP and MsmX among the *Bacillaceae* family was investigated. FrlP seems to be restricted to the *B. subtilis* group, while MsmX is phylogenetically well distributed in the *Bacillaceae* family tree, and MsmX duplication events seem to be enriched on the *B. cereus* group (Fig. 5). Regarding our data about the ecological classification of the *B. subtilis* group and other *Bacillaceae* family members that contain duplications of MsmX, it should be noted that the isolation source of a given organism may not correspond to the preferred environments of a given species; nevertheless, it can give us hints regarding possible ecological niches (56). The presence of FrlP and the respective Frl components of the fructosamines operon in the *B. subtilis* group, together with the identification of other soil organisms that can degrade fructosamines, suggests that this trait is conserved among organisms that are found associated to plants and soil, highlighting their nutritional relevance and contribution to bacterial fitness.

## MATERIALS AND METHODS

### DNA manipulation and sequencing

Regular DNA manipulations were performed as previously described by Sambrook et al. (57). PCR amplifications were carried out using Phusion High-Fidelity DNA Polymerase from Thermo Fisher Scientific according to the manufacturer’s recommendations. Restriction enzymes, FastAP Thermosensitive Alkaline Phosphatase, and T4 DNA ligase used for restriction cloning purposes were purchased from Thermo Fisher Scientific and were also used according to the manufacturer’s instructions. DNAs were purified from agarose gel bands or from PCR and restriction reaction mixtures with the NZYGelpure kit (NZYtech). Plasmid DNA was extracted and purified using the NZYMiniprep kit from NZYtech. Plasmids and PCR products were verified by restriction patterns and/or Sanger sequencing at STAB VIDA (https://www.stabvida.com/). All primers and plasmid DNA used in this work are listed on Tables S3 and S4.

### Construction of *B. subtilis* strains

The generation of the markerless in-frame Δ*frlO* deletion mutant, together with the *frlRstop* mutant in the chromosome of *B. subtilis* was achieved by overlap extension PCR followed by allelic replacement using a pMAD vector as described by Arnaud et al. (58). In brief, the immediately upstream and downstream regions of the target DNA were cloned together in the pMAD vector which was integrated in the chromosome via a single recombination event, promoted by growth at a non-permissive temperature for plasmid replication. The removal of the plasmid was upheld by a second recombination event that occurred in permissive temperature without antibiotic, allowing the withdrawal and the curing of the plasmid and the substitution of the targeted allele. The allelic replacement generated an in-frame Δ*frlO* deletion of 525 bp that comprises amino acid-coding positions 121 to 295. The creation of the *frlRstop* mutation (E36stop) was obtained by overlap extension PCR using mutagenic primers (Supplementary data and Table S3), and the allelic replacement was done using the pMAD vector as described above. The *frlRstop* mutant resulted from the exchange of codon GAA (glutamic acid) to TAA (stop codon) and was confirmed by Sanger sequencing. The *frlRstop* mutation was introduced in WT and Δ*msmX::cat* strains. Transcriptional and translational fusions of the gene *lacZ* of *E. coli* to the 5′ region of *frlP* were created by a single recombinational event at the respective locus of *B. subtilis* chromosome using integrative plasmids that contained about 340 bp of the upstream region of *frlP* plus the respective 5*′frlP-lacZ* fusions, with and without the *frlRstop* mutation. Transformation of the constructed *B. subtilis* strains was performed according to the methodology described by Anagnostopoulos and Spizizen (59). All *B. subtilis* strains used and constructed in this work are listed in Table 1. A detailed description of the construction of *B. subtilis* strains is found in the Supplementary Data section; primers and plasmids are listed in Tables S3 and S4, respectively.

**TABLE 1:** List of strains used and constructed for this work

Strain	Relevant genome	Source
*E. coli*		
XL10 Gold	Tet^r^Δ (*mcrA*)*183* Δ (*mcrCB-hsdSMR-mrr*)*173 endA1 supE44 thi-1 recA1 gyrA96 relA1 lac* Hte [F´ *proAB lacI*^q^ZΔ*M15* Tn*10* (Tet^r^) Amy Cam^r^]	Stratagene
BTH101	F-, *cya-99, araD139, galE15, galK16, rpsL1 (Str r), hsdR2, mcrA1, mcrB1*	Euromedex
*B. subtilis*		
168 T^+^	Prototroph	E. Young
IQB495	Δ*msmX*::*cat*	(11)
IQB618	Δ*frlP*(*yurJ*)	(12)
IQB634	Δ*frlP*(*yurJ*) Δ*msmX*::*cat*	(60)
ISN71	*frlRstop36*	pIG6→168T^+^
ISN88	Δ*frlO*	pIT1→168T^+^
ISN128	Φ(*frlP*’*-lacZ*+) (trancriptional fusion) *km*	pIG25→168T^+^
ISN129	Φ(*frlP*’*-lacZ*+) (translational fusion) *km*	pIG26→168T^+^
ISN130	*frlRstop36* Φ(*frlP*’*-lacZ*+) (trancriptional fusion) *km*	pIG27→ISN71
ISN131	*frlRstop36* Φ(*frlP*’*-lacZ*+) (translational fusion) *km*	pIG28→ISN71

### Growth conditions

*Escherichia coli* XL10-Gold (Stratagene), used for the construction of all plasmids, and *E. coli* BTH101, used for B2H assays, were all grown in liquid Lysogeny Broth (LB) medium (61) and on LB solidified with 1.6% (wt/vol) agar. LB media were supplemented with ampicillin (100 µg mL^−1^), kanamycin (30 µg mL^−1^), tetracycline (12 µg mL^−1^), IPTG (0.1 or 0.5 mM), or streptomycin (100 µg mL^−1^) when appropriate.

*B. subtilis* strains were routinely grown in liquid LB medium, LB solidified with 1.6% (wt/vol) agar or liquid SP medium (62), supplemented with chloramphenicol (5 µg mL^−1^), kanamycin (10 µg mL^−1^), erythromycin (1 µg mL^−1^), and/or X-Gal (80 µg mL^−1^), when suitable. Growth kinetic parameters of *B. subtilis* were determined in M9 minimal medium (61) without trace elements, supplemented with 1 mM D-(+)-glucose (Sigma), 1 mM D(−) fructose (Merck), and 2 mM fructosevaline (mixture of diastereomers) (CymitQuimica). Starter cultures were grown overnight in 5 mL M9 minimal medium supplemented with 27.8 mM glucose and 0.05% (wt/vol) Casein acid hydrolysate, from bovine milk (CH) (Fluka) and diluted to an initial OD_600nm_ of 0.01 in 200 µL M9 minimal medium supplemented with 22 µg mL^−1^ CAF and with the respective carbon and nitrogen source on a 96 well plate. Growth was followed by measuring the optical density (OD_600nm_) in 15 min intervals for 12 h or 48 h (depending on the carbon source) in a microplate reader (Spectramax) at 37°C with agitation. Growth curves were analyzed after normalizing the OD_600nm_ with blank absorbance values and were used to determine the doubling time of each strain when growth phenotype was observed. Doubling time was calculated with absorbance values taken between 1 h and 3 h before each growth peak.

### Bacterial adenylate cyclase two-hybrid (B2H) system

The bacterial adenylate cyclase two-hybrid system ([31]; Euromedex) was used to test protein-protein interactions between nucleotide-binding domains (NBDs) MsmX and FrlP and transmembrane domains (TMDs) FrlN and FrlM. ATPases were fused to fragment T18 in the pUT18 vector, while TMDs were fused to fragment T25 in pKT25 or pKNT25 plasmids. Construction of plasmids for B2H is described in the Supplementary Data section. *E. coli* BTH101 was co-transformed with a combination of one pUT18 and one pKT25 or pKNT25 derivatives; the resulting co-transformants were selected in LA supplemented with the appropriate antibiotics. Co-transformants were inoculated in LB with the respective antibiotics and IPTG (0.5 mM), grown aerobically at 30°C and protein-protein interactions were assayed for β-galactosidase activity following kit instructions. Interactions were also assessed by spotting 3 µL of overnight cultures onto LB agar plates supplemented with the appropriate antibiotics, IPTG (0.5 mM), and X-Gal (40 µg mL⁻¹). Plates were incubated at 30°C for 24 h prior to analysis.

### β-Galactosidase assays of *B. subtilis* strains

*B. subtilis* strains holding transcriptional and translational fusions of the 5′ region of *frlP* to *lacZ* gene were grown in M9 minimal medium in a 96 well plate supplemented with CAF plus glucose, fructose, or fructosevaline, as described above, or 1% (wt/vol) casein acid hydrolysate from bovine milk (CH) (Fluka). Two hundred microliters of each triplicate (600 µL total) was recovered to the same microcentrifuge tube at the approximate growth peak of each strain and kept at −20°C until the β-galactosidase assay was performed the next day. Cells were resuspended in 1 mL Z buffer (61) with 100 µg mL^−1^ lysozyme and left incubating at 37°C for 5 min. Triton X-100 0.1% (wt/vol) was added to each tube, and the cells were vigorously vortexed for 10 s and incubated on ice for 5 min (adapted from reference 63). The tubes were incubated at 28°C for 10 min in a dry bath before starting the assay. β-Galactosidase activity was measured and expressed in Miller units (M.U.) as previously described, using the substrate ONPG (61). *B. subtilis* 168T^+^ (WT) (without *lacZ* fusions) was used as control; M.U. range between 1.4 and 2.7 in glucose, fructose, and CH and between 5.1 and 12 in fructosevaline.

### Phylogenomic reconstruction of the *Bacillaceae*

A total of 629 reference genomes and respective proteomes from the *Bacillaceae* family and the outgroup *Staphylococcus aureus* were retrieved from NCBI (accessed on 23 January 2023, Table S1). These proteomes were used as input for Orthofinder v2.5.4 (64) which inferred orthogroups based on all-versus-all sequence similarity searches using DIAMOND, followed by multiple sequence alignment with MAFFT and gene trees using IQ-TREE (65). A species phylogeny was reconstructed from 100 orthogroups that contained single-copy genes in at least 48.6% of species. The resulting phylogeny was rooted using *Staphylococcus aureus*.

### HMM-based search of FrlP and MsmX across *Bacillaceae* and respective genomic context

To search for MsmX and FrlP across all species, an HMM profile was first constructed. For that the reviewed protein sequences of MsmX (P94360) and FrlP (O32151) from *B. subtilis* were retrieved from UniProt and used as queries for a BLASTp search in NCBI refseq database. Hits with an e-value below 0.001 were retained, up to a maximum of 100 hits per protein. These sequences were then aligned using MAFFT v7.407 (66), and HMM profiles for each gene were generated using HMMER v3.3.3 (67) within Orthofisher (36). The resulting HMM profiles were used to assess presence/absence of each gene and respective copy number using Orthofisher v1.0.5 (36) with default parameters. Given the high homology between the two proteins (MsmX and FrlP), we further confirmed orthology of the identified hits by Orthofisher by constructing a phylogeny (Fig. S4). For that, all the identified protein sequences by Orthofisher (both MsmX and FrlP) were aligned with MAFFT and used to construct a phylogenetic tree with IQ-TREE. Absence of FrlP was confirmed by BLASTp searches against the proteomes of the following species: *B. haynesii*, *B. paralicheniformis*, *B. licheniformis*, *B. swezeyi*, *B. australimaris*, *B. safensis*, *B. pumilus*, *B. zhangzhouensis*, *B. stratosphericus*, *B. gobiensis*, *B. capparidis*, *M. crassostreae*, *M. mangrovi*, *D. indicus*, *N. cucumis*, *S. horikoshii,* and *G. kaustophilus*. The best hits were then used in a reciprocal BLASTp in NCBI against *B. subtilis* 168. *B. velezensis*, *B. sonorensis,* and *B. amyloliquefaciens* were used as positive controls for the presence of these genes. Additionally, in order to confirm that absences were not originated by annotation issues, tBLASTx searches were performed for *B. haynesii*, *B. safensis*, *B. stratosphericus*, and *M. mangrovi* against the respective genome assemblies. These analyses confirmed the absence of FrlP in all analyzed species. The same procedure was conducted for *msmX*, for which tBLASTx searches were performed against the genome assemblies of 18 species identified as either lacking *msmX* or containing only a single copy. The results pointed to the presence of *msmX* in 15 species. Therefore, it is important to note that the methodology employed may result in an underestimation of *msmX* presence. For species in which the presence of FrlP was confirmed and for representative species containing multiple MsmX copies, the genomic context was further analyzed. For this, the complete proteome was predicted using AUGUSTUS v3.3.3 (68), with the complete gene model and *Staphylococcus aureus* as reference. From the AUGUSTUS output, coordinates for the genes of interest were extracted and used to generate genomic plots in R using the genoPlotR package.

## SUPPLEMENTAL MATERIAL

Supplemental materials jb.00320-25-s0001.docx Additional experimental details, Figures S1 to S4 and Tables S2 to S4.

Table S1 jb.00320-25-s0002.xlsx List of species analyzed.

ASM does not own the copyrights to Supplemental Material that may be linked to, or accessed through, an article. The authors have granted ASM a non-exclusive, world-wide license to publish the Supplemental Material files. Please contact the corresponding author directly for reuse.
