# Supplementary material for: FrlP, an ABC type I importer component of Bacillus subtilis: regulation and impact in bacterial fitness
Source: J Bacteriol. 2025 Oct 30;207(11):e00320-25. doi: 10.1128/jb.00320-25 (PMC12632257; doi:10.1128/jb.00320-25)
Supplement: Supplemental materials — Additional experimental details, Figures S1 to S4 and Tables S2 to S4. [file jb.00320-25-s0001.docx]

## Supplementary Data

FrlP, an ABC type I importer component of *Bacillus subtilis*: regulation and impact in bacterial fitness

Inês C. Gonçalves,^a,c^ Ana Pontes,^b,c^ Carla Gonçalves,^b,c^ Isabel de Sá Nogueira^a,c^#

^a^ Microbial Genetics Laboratory, UCIBIO, Department of Life Sciences, NOVA School of Science and Technology, Universidade NOVA de Lisboa, Caparica, Portugal.

^b^ Yeast Genomics Laboratory, UCIBIO, Department of Life Sciences, NOVA School of Science and Technology, Universidade NOVA de Lisboa, Caparica, Portugal.

^c^ Associate Laboratory i4HB, NOVA School of Science and Technology, Universidade NOVA de Lisboa, Caparica, Portugal

**Table S2** Ecological distribution of deposited genomes on NCBI (BioSamples) of *Bacillaceae* family members that contain FrlP or more than one copy of MsmX. The first two rows represent the number of copies of MsmX and FrlP in the respective organism. The niche that is more representative for each organism is highlighted in lighter grey.

* *Bacillus subtilis* sensus stricto BioSamples were not analyzed because it is well established as a soil and gastrointestinal commensal organism. It is an easily dispersible and persistent organism and, therefore, several Biosample entries are available.

| MsmX | FrlP | Organism | Total entries | Soil and Plants | Foods and beverages | Host-associated | Aquatic | Other | Unidentified /missing (%) |  |
| --- | --- | --- | --- | --- | --- | --- | --- | --- | --- | --- |
| FrlP presence | | | | | | | | | | |
| 1 | 1 | *Bacillus amyloliquefaciens* | *996* | 318 | 65 | 67 | 11 | 49 | 48.80% |  |
|  |  |  |  | 31.93% | 6.53% | 6.73% | 1.10% | 4.92% |  |  |
| 1 | 1 | *Bacillus atrophaeus subsp. globigii* | 4 | 1 |  |  |  |  | 75.00% |  |
|  |  |  |  | 25.00% | 0.00% | 0.00% | 0.00% | 0.00% |  |  |
| 1 | 1 | *Bacillus cabrialesii* | 5 | 5 |  |  |  |  | 0.00% |  |
|  |  |  |  | 100.00% | 0.00% | 0.00% | 0.00% | 0.00% |  |  |
| 1 | 1 | *Bacillus glycinifermentans* | 33 | 6 | 3 | 17 |  |  | 21.21% |  |
|  |  |  |  | 18.18% | 9.09% | 51.52% | 0.00% | 0.00% |  |  |
| 1 | 1 | *Bacillus halotolerans* | 92 | 58 | 10 | 3 | 4 |  | 18.48% |  |
|  |  |  |  | 63.04% | 10.87% | 3.26% | 4.35% | 0.00% |  |  |
| 1 | 1 | *Bacillus inaquosorum* | 141 | 31 | 7 |  |  |  | 73.05% |  |
|  |  |  |  | 21.99% | 4.96% | 0.00% | 0.00% | 0.00% |  |  |
| 1 | 1 | *Bacillus mojavensis* | 52 | 32 |  | 2 | 2 |  | 30.77% |  |
|  |  |  |  | 61.54% | 0.00% | 3.85% | 3.85% | 0.00% |  |  |
| 1 | 1 | *Bacillus nakamurai* | 5 | 4 |  |  |  | 1 | 0.00% |  |
|  |  |  |  | 80.00% | 0.00% | 0.00% | 0.00% | 20.00% |  |  |
| 1 | 1 | *Bacillus rugosus* | 3 | 2 |  | 1 |  |  | 0.00% |  |
|  |  |  |  | 66.67% | 0.00% | 33.33% | 0.00% | 0.00% |  |  |
| 1 | 1 | *Bacillus siamensis* | 57 | 30 | 5 | 5 | 3 | 2 | 21.05% |  |
|  |  |  |  | 52.63% | 8.77% | 8.77% | 5.26% | 3.51% |  |  |
| 1 | 1 | *Bacillus sonorensis* | 71 | 12 | 8 | 34 |  | 2 | 21.13% |  |
|  |  |  |  | 16.90% | 11.27% | 47.89% | 0.00% | 2.82% |  |  |
| 1 | 1 | *Bacillus stercoris* | 22 | 8 | 2 | 4 |  | 5 | 13.64% |  |
|  |  |  |  | 36.36% | 9.09% | 18.18% | 0.00% | 22.73% |  |  |
| 1 | 1 | *Bacillus subtilis** | 9247 |  |  |  |  |  |  |  |
| 1 | 1 | *Bacillus subtilis subsp. spizizenii* | 305 | 48 |  | 2 | 4 | 91 | 52.46% |  |
|  |  |  |  | 15.74% | 0.00% | 0.66% | 1.31% | 29.84% |  |  |
| 1 | 1 | *Bacillus tequilensis* | 21 | 9 | 1 | 6 | 2 |  | 14.29% |  |
|  |  |  |  | 42.86% | 4.76% | 28.57% | 9.52% | 0.00% |  |  |
| 1 | 1 | *Bacillus vallismortis* | 77 | 12 | 2 |  | 1 | 51 | 14.29% |  |
|  |  |  |  | 15.58% | 2.60% | 0.00% | 1.30% | 66.23% |  |  |
| 1 | 1 | *Bacillus velezensis* | 1604 | 675 | 157 | 140 | 47 | 159 | 26.56% |  |
|  |  |  |  | 42.08% | 9.79% | 8.73% | 2.93% | 9.91% |  |  |
| MsmX duplications | | | | | | | | | | |
| 2 | 0 | *Alkalihalobacillus shacheensis* | 1 | 1 |  |  |  |  | 0.00% |  |
|  |  |  |  | 100.00% | 0.00% | 0.00% | 0.00% | 0.00% |  |  |
| 3 | 0 | *Aquibacillus albus* | 1 |  |  |  |  |  | 100.00% |  |
|  |  |  |  | 0.00% | 0.00% | 0.00% | 0.00% | 0.00% |  |  |
| 2 | 0 | Bacillus albus | 43 | 12 |  | 6 | 13 | 10 | 4.65% |  |
|  |  |  |  | 27.91% | 0.00% | 13.95% | 30.23% | 23.26% |  |  |
| 2 | 0 | Bacillus alveayuensis | 4 |  |  |  | 3 |  | 25.00% |  |
|  |  |  |  | 0.00% | 0.00% | 0.00% | 75.00% | 0.00% |  |  |
| 2 | 0 | Bacillus capparidis | 3 | 1 |  |  |  |  | 66.67% |  |
|  |  |  |  | 33.33% | 0.00% | 0.00% | 0.00% | 0.00% |  |  |
| 2 | 0 | Bacillus clarus | 2 | 2 |  |  |  |  | 0.00% |  |
|  |  |  |  | 100.00% | 0.00% | 0.00% | 0.00% | 0.00% |  |  |
| 2 | 0 | Bacillus dafuensis | 3 | 1 |  | 1 |  | 1 | 0.00% |  |
|  |  |  |  | 33.33% | 0.00% | 33.33% | 0.00% | 33.33% |  |  |
| 2 | 0 | Bacillus gaemokensis | 2 |  |  |  | 2 |  | 0.00% |  |
|  |  |  |  | 0.00% | 0.00% | 0.00% | 100.00% | 0.00% |  |  |
| 3 | 0 | Bacillus luti | 11 | 2 |  | 5 | 3 |  | 9.09% |  |
|  |  |  |  | 18.18% | 0.00% | 45.45% | 27.27% | 0.00% |  |  |
| 2 | 0 | Bacillus manliponensis | 1 |  |  |  | 1 |  | 0.00% |  |
|  |  |  |  | 0.00% | 0.00% | 0.00% | 100.00% | 0.00% |  |  |
| 3 | 0 | Bacillus mobilis | 66 | 8 | 7 | 17 | 2 | 14 | 27.27% |  |
|  |  |  |  | 12.12% | 10.61% | 25.76% | 3.03% | 21.21% |  |  |
| 2 | 0 | Bacillus nitratireducens | 27 | 13 | 1 | 3 | 2 |  | 29.63% |  |
|  |  |  |  | 48.15% | 3.70% | 11.11% | 7.41% | 0.00% |  |  |
| 3 | 0 | Bacillus pacificus | 119 | 13 | 39 | 25 | 7 | 3 | 26.89% |  |
|  |  |  |  | 10.92% | 32.77% | 21.01% | 5.88% | 2.52% |  |  |
| 3 | 0 | Bacillus paramycoides | 76 | 15 |  | 1 | 2 | 38 | 26.32 |  |
|  |  |  |  | 19.74% | 0.00% | 1.32% | 2.63% | 50.00% |  |  |
| 2 | 0 | Bacillus paranthracis | 348 | 44 | 114 | 94 | 6 | 19 | 20.40% |  |
|  |  |  |  | 12.64% | 32.76% | 27.01% | 1.72% | 5.46% |  |  |
| 2 | 0 | Bacillus proteolyticus | 12 | 5 |  |  | 2 |  | 41.67% |  |
|  |  |  |  | 41.67% | 0.00% | 0.00% | 16.67% | 0.00% |  |  |
| 4 | 0 | Bacillus pseudomycoides | 147 | 125 | 1 | 4 |  |  | 11.56% |  |
|  |  |  |  | 85.03% | 0.68% | 2.72% | 0.00% | 0.00% |  |  |
| 2 | 0 | Bacillus rhizoplanae | 1 |  |  |  |  |  | 100.00% |  |
|  |  |  |  | 0.00% | 0.00% | 0.00% | 0.00% | 0.00% |  |  |
| 2 | 0 | Bacillus testis | 2 |  |  | 1 |  |  |  |  |
|  |  |  |  | 0.00% | 0.00% | 50.00% | 0.00% | 0.00% | 50.00% |  |
| 2 | 0 | Bacillus wiedmannii | 258 | 147 | 35 | 26 | 6 | 3 | 15.89% |  |
|  |  |  |  | 56.98% | 13.57% | 10.08% | 2.33% | 1.16% |  |  |
| 2 | 0 | Bacillus xiapuensis | 2 | 2 |  |  |  |  | 0.00% |  |
|  |  |  |  | 100.00% | 0.00% | 0.00% | 0.00% | 0.00% |  |  |
| 2 | 0 | Fictibacillus nanhaiensis | 8 | 1 |  | 2 | 3 | 1 | 12.50% |  |
|  |  |  |  | 12.50% | 0.00% | 25.00% | 37.50% | 12.50% |  |  |
| 2 | 0 | Lederbergia citri | 1 | 1 |  |  |  |  | 0.00% |  |
|  |  |  |  | 100.00% | 0.00% | 0.00% | 0.00% | 0.00% |  |  |
| 2 | 0 | Litchfieldia alkalitelluris | 2 | 2 |  |  |  |  | 0.00% |  |
|  |  |  |  | 100.00% | 0.00% | 0.00% | 0.00% | 0.00% |  |  |
| 2 | 0 | Lysinibacillus halotolerans | 2 | 1 |  |  |  |  | 50.00% |  |
|  |  |  |  | 50.00% | 0.00% | 0.00% | 0.00% | 0.00% |  |  |
| 2 | 0 | Lysinibacillus telephonicus | 2 |  | 1 |  |  | 1 | 0.00% |  |
|  |  |  |  | 0.00% | 50.00% | 0.00% | 0.00% | 50.00% |  |  |
| 2 | 0 | Lysinibacillus timonensis | 1 |  |  | 1 |  |  | 0.00% |  |
|  |  |  |  | 0.00% | 0.00% | 100.00% | 0.00% | 0.00% |  |  |
| 2 | 0 | Mesobacillus persicus | 1 |  |  |  |  |  | 100.00% |  |
|  |  |  |  | 0.00% | 0.00% | 0.00% | 0.00% | 0.00% |  |  |
| 2 | 0 | Oceanobacillus chungangensis | 1 |  |  |  | 1 |  | 0.00% |  |
|  |  |  |  | 0.00% | 0.00% | 0.00% | 100.00% | 0.00% |  |  |
| 2 | 0 | Oceanobacillus polygoni | 3 |  | 2 |  |  |  | 33.33% |  |
|  |  |  |  | 0.00% | 66.67% | 0.00% | 0.00% | 0.00% |  |  |
| 2 | 0 | Oceanobacillus rekensis | 1 | 1 |  |  |  |  | 0.00% |  |
|  |  |  |  | 100.00% | 0.00% | 0.00% | 0.00% | 0.00% |  |  |
| 2 | 0 | Oceanobacillus zhaokaii | 1 |  |  | 1 |  |  | 0.00% |  |
|  |  |  |  | 0.00% | 0.00% | 100.00% | 0.00% | 0.00% |  |  |
| 2 | 0 | Peribacillus simplex | 72 | 43 | 2 | 3 |  | 14 | 13.89% |  |
|  |  |  |  | 59.72% | 2.78% | 4.17% | 0.00% | 19.44% |  |  |
| 2 | 0 | Pontibacillus litoralis | 1 |  |  | 1 |  |  | 0.00% |  |
|  |  |  |  | 0.00% | 0.00% | 100.00% | 0.00% | 0.00% |  |  |
| 2 | 0 | Priestia aryabhattai | 156 | 81 | 3 | 8 | 6 | 32 | 16.67% |  |
|  |  |  |  | 51.92% | 1.92% | 5.13% | 3.85% | 20.51% |  |  |
| 3 | 0 | Priestia endophytica | 20 | 9 |  | 5 |  | 2 | 20.00% |  |
|  |  |  |  | 45.00% | 0.00% | 25.00% | 0.00% | 10.00% |  |  |
| 2 | 0 | Priestia filamentosa | 15 | 9 |  |  | 2 | 1 | 20.00% |  |
|  |  |  |  | 60.00% | 0.00% | 0.00% | 13.33% | 6.67% |  |  |
| 3 | 0 | Priestia megaterium | 966 | 639 | 8 | 52 | 21 | 75 | 17.70% |  |
|  |  |  |  | 66.15% | 0.83% | 5.38% | 2.17% | 7.76% |  |  |
| 2 | 0 | Psychrobacillus lasiicapitis | 2 |  |  | 1 |  |  | 50.00% |  |
|  |  |  |  | 0.00% | 0.00% | 50.00% | 0.00% | 0.00% |  |  |
| 2 | 0 | Salibacterium aidingense | 2 |  |  |  | 1 |  | 50.00% |  |
|  |  |  |  | 0.00% | 0.00% | 0.00% | 50.00% | 0.00% |  |  |
| 2 | 0 | Salibacterium salarium | 2 | 1 |  |  |  |  | 50.00% |  |
|  |  |  |  | 50.00% | 0.00% | 0.00% | 0.00% | 0.00% |  |  |
| 2 | 0 | Sediminibacillus dalangtanensis | 1 | 1 |  |  |  |  | 0.00% |  |
|  |  |  |  | 100.00% | 0.00% | 0.00% | 0.00% | 0.00% |  |  |
| 2 | 0 | Sutcliffiella halmapala | 1 | 1 |  |  |  |  | 0.00% |  |
|  |  |  |  | 100.00% | 0.00% | 0.00% | 0.00% | 0.00% |  |  |
| 3 | 0 | Terrilactibacillus laevilacticus | 3 | 3 |  |  |  |  | 0.00% |  |
|  |  |  |  | 100.00% | 0.00% | 0.00% | 0.00% | 0.00% |  |  |
| 3 | 0 | Terrilactibacillus tamarindi | 1 | 1 |  |  |  |  | 0.00% |  |
|  |  |  |  | 100.00% | 0.00% | 0.00% | 0.00% | 0.00% |  |  |

**Table S3** List of oligonucleotides used in this work; modified nucleotides for point mutations and restriction sites are underlined.

| Oligonucleotide | Sequence (5’ - 3’) |
| --- | --- |
| ARA583 | TCGCGGTTTCGCTGCCCTTT |
| ARA584 | AAGTCCCGCAACGCGCGCAA |
| ARA826 | CAAAAAGGATCTTCACCTAGATCC |
| ARA871 | ATTATTGATGGATCCGTGCCG |
| ARA872 | GTTTGATGAATTCAGCTGACG |
| ARA873 | CGCTTCTGAAGATCCTTTGGTGTCATTTTTGTAGTAGG |
| ARA874 | CCTACTACAAAAATGACACCAAAGGATCTTCAGAAGCG |
| ARA916 | GGAATTCGGTCTGGTCATGC |
| ARA919 | CGGGATCCGCTGCATACGAGCG |
| ARA925 | CGCCGAAAGAACGTGATATT |
| ARA926 | TCGCCATCTTTCTGAGCTTT |
| ARA929 | GCTCGTTTTAGGTAGGCAGC |
| ARA930 | GCTGCCTACCTAAAACGAGC |
| ARA932 | GGGAATTGCTTCTAGACTCAATATAAG |
| ARA933 | GGTTTTTTTGCTCTAGACGGATTCTCAC |
| ARA943 | GTTGTTTCTAGAGACGAAATAACGGG |
| ARA944 | GGAGCACCTTTTCTAGATACACAGCC |
| ARA945 | GTTTCCTCCTTGGTACCCCTTTGACTC |
| ARA951 | TTAAGGGGGAGGATCCAATGTTGCGG |
| ARA1030 | AATCTAGAGTGAAGCTTCGCACC |
| ARA1031 | ATTCTAGATACTCCTCCTTTCCGG |
| ARA1033 | CCGGTACCCCTTTTACACTGCCG |
| ARA1050 | GATCTAGAGATGCTGCCTCAGAAG |
| ARA1051 | ATTTCGCGAACGGGCAGACATGGC |
| ARA1052 | CAGAATTCCTGACGGTGATGCG |
| ARA1053 | AACCCGGGTCGACGTGTTCAAATGTTAATGAAGCC |

**Table S4** List of plasmids used in this work.

| Plasmid | Relevant Construction | Source or reference |
| --- | --- | --- |
| pIG20 | pKNT25 derivative for the expression of FrlN-T25 fusion protein, *kan* | This work |
| pIG23 | pKT25 derivative for the expression of T25-FrlM fusion protein, *kan* | This work |
| pIG24 | Integrative plasmid with a *lacZ* gene for the construction of transcriptional and translational fusions, *kan* | This work |
| pIG25 | Integrative plasmid used for the construction of *in situ* 5’*frlP*-*lacZ* (Φ(*frlP*’*-lacZ*+)) transcriptional fusion, *kan* | This work |
| pIG26 | Integrative plasmid used for the construction of *in situ* 5’*frlP*-*lacZ* (Φ(*frlP*’*-lacZ*+))translational fusion, *kan* | This work |
| pIG27 | Integrative plasmid used for the construction of *in situ* *frlRstop* 5’*frlP*-*lacZ* (Φ(*frlP*’*-lacZ*+)) transcriptional fusion, *kan* | This work |
| pIG28 | Integrative plasmid used for the construction of *in situ frlRstop* 5’*frlP*-*lacZ* (Φ(*frlP*’*-lacZ*+)) translational fusion, *kan* | This work |
| pIG6 | pMAD derivative used for the introduction of the nonsense mutation in *frlR* GAA (Glu at position 36) to TAA (stop codon), *bla*, *erm* | This work |
| pIT1 | pMAD derivative used for Δ*frlO*, *bla*, *erm* | This work |
| PJL3 | pLitmus29 derivative used as a source of kanamycin resistance cassette, *bla*, *kan* | (3) |
| pJM783 | Integrative vector containing *lacZ*, *bla*, *cat* | (2) |
| pJS1 | pUT18 derivative for the expression of FrlP-T18 fusion protein, *bla* | This work |
| pKNT25 | B2H expression vector for N-terminal fusions to T25 fragment of CyaA, *kan* | (5) |
| pKT25 | B2H expression vector for C-terminal fusions to T25 fragment of CyaA, *kan* | (5) |
| pKT25-zip | pUKT25 derivative with zip fused to T25 fragment used as positive control for B2H, *bla* | (5) |
| pLG55 | pUT18 derivative for the expression of MsmX-T18 fusion protein, *bla* | This work |
| pLG64 | pKT25 derivative for the expression of T25-AraQ fusion protein, *kan* | This work |
| pMAD | Plasmid used for allelic replacement in Gram-positive bacteria, *bla*, *erm* | (1) |
| pUT18 | B2H expression vector for N-terminal fusions to T18 fragment of CyaA, *bla* | (5) |
| pUT18-zip | pUT18 derivative with zip fused to T18 fragment used as positive control for B2H, *bla* | (5) |

**Supplementary figures**


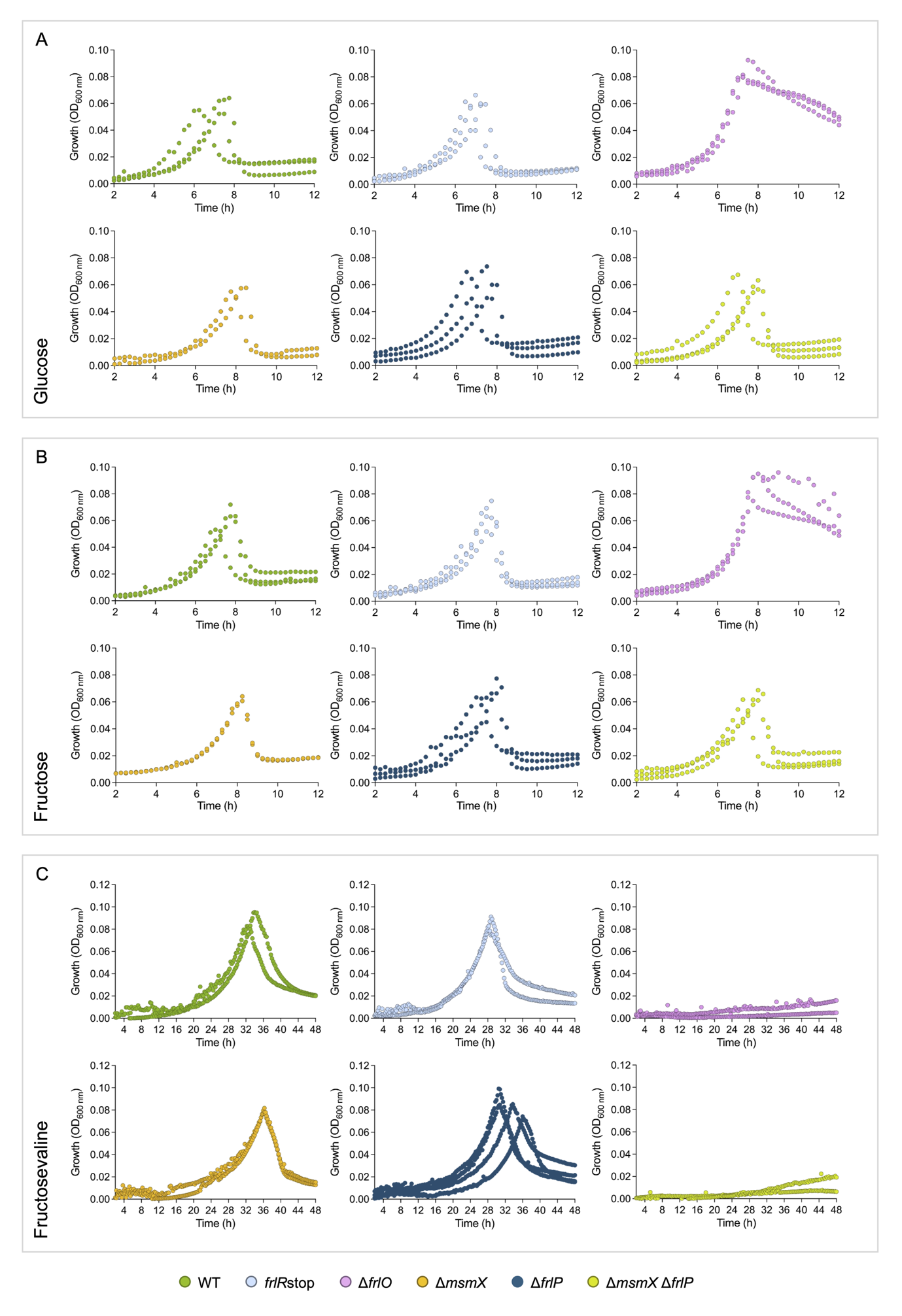


**Fig. S1.** Growth analysis of *B. subtilis* WT (darker green), *frlRstop* (light blue), Δ*frlO* (pink), Δ*msmX* (orange), Δ*frlP* (dark blue) and Δ*msmX*Δ*frlP* (light green) strains in M9 minimal medium supplemented with 22 μg mL^-1^ CAF and 1 mM glucose **(A)**, 1 mM fructose **(B)** or 2 mM fructosevaline **(C)**. At least two biological replicates are shown, each resulting from the mean of three technical replicates.


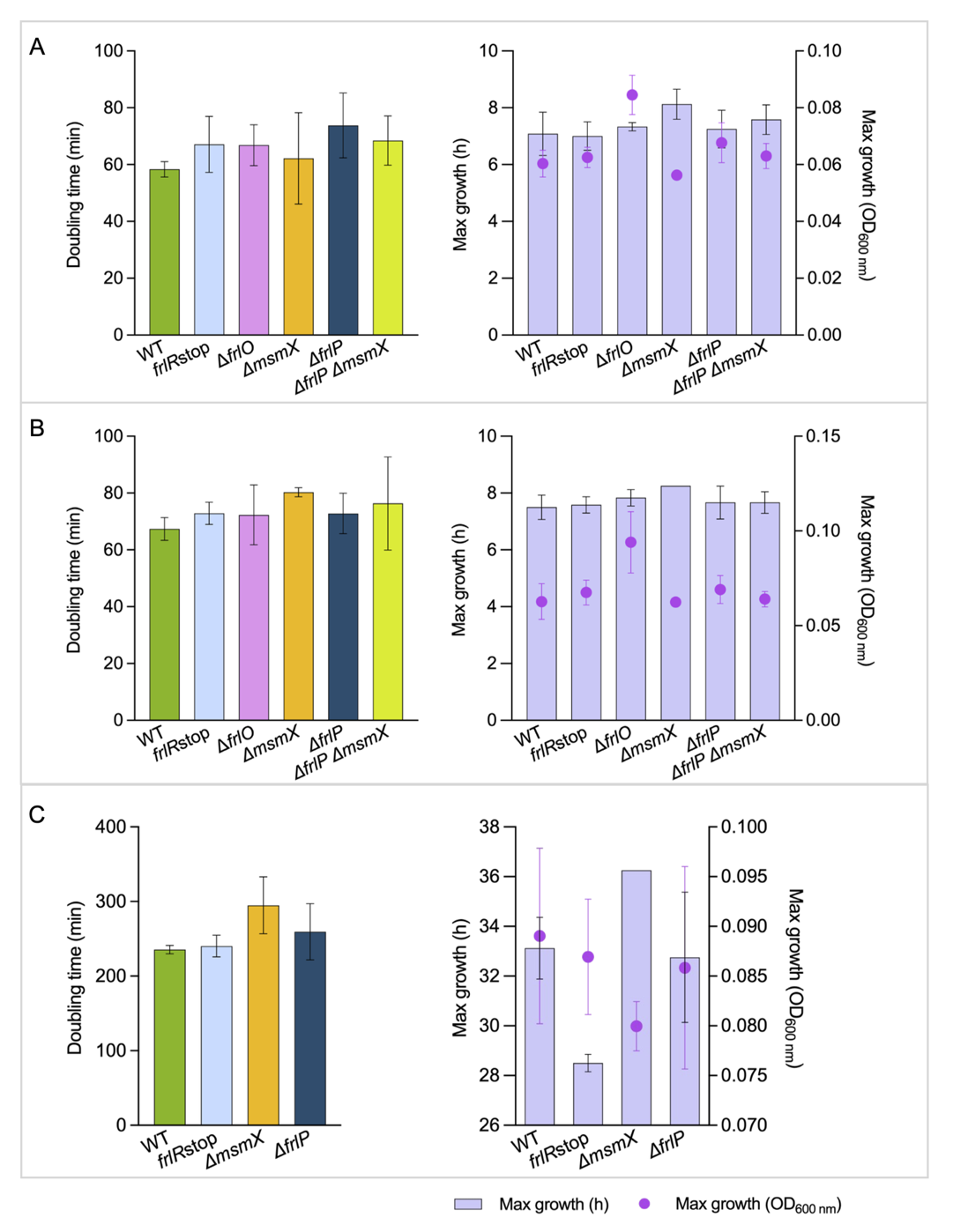


**Fig. S2.** Growth kinetic parameters of *B. subtilis* strains grown in M9 minimal medium supplemented with 22 μg mL^-1^ CAF and 1 mM glucose **(A)**, 1 mM fructose **(B)** or 2 mM fructosevaline **(C)**, from growth analysis shown in Figure S1. Left panels represent doubling time in minutes. Right panels represent maximum growth values, in hours of growth (bars) and in maximum OD_600nm_ values obtained (dots). Error bars represent standard deviation of the mean from at least two independent growth analyses.

**Fig. S3.** Maximum likelihood phylogenomic tree comprising 629 species from the *Bacillaceae* family inferred from the concatenated alignment of 100 single-copy orthogroups and rooted with *Staphylococcus aureus*. The species from the *Bacillus subtilis* and *Bacillus cereus* groups are highlighted. Presence/absence of *msmX*/*frlP* and number of protein-coding genes are depicted in the ring area.

**Fig. S4.** Maximum likelihood phylogeny comprising the protein sequences retrieved from Orthofisher of MsmX and FrlP, depicted in orange and green, respectively. The reference sequences of FrlP and MsmX from *B. subtilis* are highlighted in **bold**.

# Construction of plasmids and *Bacillus subtilis* strains

The construction of markerless Δ*frlO* and the *frlRstop* mutation in the *B. subtilis* chromosome was obtained by allelic replacement using the pMAD vector preceded by overlap PCR, as described by Arnaud *et al.* (1). For deletion purposes primers were designed to amplify the upstream and downstream regions of the section to be deleted; mutagenic primers were used to create the nonsense point mutation in the *frlR* gene. For the construction of the Δ*frlO* mutant, chromosomal DNA of *B. subtilis* 168T^+^ was used for amplification of two PCR products with primers ARA871 and ARA873, and ARA872 and ARA874. The products from both PCR reactions were joined by overlap PCR using external primers ARA871 and ARA872; the resulting amplicon was digested with EcoRI and BamHI and cloned into pMAD digested with the same enzymes and dephosphorylated, yielding pIT1. The *frlRstop* construct was made by overlap extension PCR using external primers ARA916 and ARA919, and mutagenic primers ARA929 and ARA930. The mutagenic primers introduced the E36stop mutation in *frlR* by exchanging the glutamic acid encoding-codon (GAA) to a nonsense mutation (TAA). The overlap fragment was subcloned between the EcoRI and BamHI sites of pMAD (1), yielding pIG6. pIT1 and pIG6 were integrated in the *B. subtilis* chromosome by a single recombination event, forced by growth with the appropriate antibiotic at 42ºC, for which the plasmid is non replicative. A second recombination event was promoted by growth at a permissive temperature without antibiotic, resulting in the restoration of the genotype or the clean introduction of the desired alteration. The detailed procedure for the generation of these clean alterations is described in detail by Arnaud *et al.*, 2004 (1). The in-frame Δ*frlO* construction resulted in the removal of a 525 bp region between amino acid-encoding positions 121 and 295, generating the *B. subtilis* strain ISN88. The *frlRstop* mutation was introduced in a WT and in a Δ*msmX* background, yielding strains ISN71 and ISN72, respectively.

The construction of *B. subtilis* with 5’*frlP*-*lacZ* transcriptional and translational fusions in WT and *frlRstop* backgrounds was caried out by placing a *lacZ* gene copy of *E. coli* fused to the 5’-region of *frlP* using integrative plasmids. For these constructions pIG24, pIG25, pIG26, pIG27 and pIG28 were assembled. pIG24 is the backbone of the remaining vectors and was created by subcloning the *lacZ* region from pJM783 (2) into pJL3 (3) between KspAI and EcoRI sites. The *lacZ* gene was amplified by PCR with primers ARA826 and ARA1051 and treated with EcoRI and Bsp68I before cloning. For the construction of the remaining plasmids, the 5’ region of *frlP* was amplified by PCR from chromosomal DNA of *B. subtilis* 168T^+^ with primers ARA1052 and ARA1053, which contain additional EcoRI and SmaI/SalI restriction sites, respectively. The resulting fragments of approximately 378 bp were digested with EcoRI and SmaI for transcriptional fusions or with EcoRI and SalI for translational fusions. The products were cloned into pIG24 treated with the respective set of restriction enzymes, yielding plasmids pIG25 and pIG26 with transcriptional and translational fusions, respectively. These plasmids were used as templates for site-directed mutagenesis using mutagenic primers ARA929 and ARA930 to introduce the nonsense mutation *frlRstop* (described above), yielding pIG27 and pIG28, respectively. The fusions were introduced via transformation of *B. subtilis* 168T^+^ or *B. subtilis* ISN71 with the respective plasmids followed by a single crossover event, yielding strains ISN128 to ISN131.

All *B. subtilis* strains were transformed based on the protocol by Anagnostopoulos and Spizizen (4). All modifications were confirmed by PCR and sequencing. Strains used and constructed for this work are summarized in Table 1.

**Bacterial adenylate cyclase two-hybrid (B2H) system: construction of expression vectors for protein-protein interaction studies**

To test protein-protein interactions between nucleotide binding domains (NBDs) MsmX and FrlP and transmembrane domains (TMDs) FrlN and FrlM, the B2H System ((5); Euromedex) was used. Zip-Zip and MsmX-AraQ interactions were used as positive controls, and empty pKT25 and pUT28 were used as negative controls for interaction. The open reading frames (ORFs) of the ATPases were cloned in pUT18 vectors and the ORFs of permeases were cloned in pKT25/pKNT25 to generate in-frame fusions to the respective T18 or T25 fragments. We found that this combination of NBDs fused to T18 and TMDs fused to T25 was the one for which the interaction test was more efficient (unpublished work). The ORFs of *msmX* and *frlP* were PCR-amplified with primers ARA932 and ARA933 and ARA943 and ARA944, respectively, that introduced XbaI restriction sites on both 5’ and 3’ ends. These ORFs, treated with XbaI, were ligated to pUT18 digested with the same enzyme, yielding plasmids pLG55 and pJS1. Using primers containing additional restriction sites KpnI and BamHI, ARA945 and ARA951, *araQ* was amplified by PCR, treated with KpnI and BamHI and cloned into pKT25 similarly digested, yielding pLG64. Primers ARA1030 and ARA1031, containing XbaI restriction sites, were used for the PCR amplification of *frlN*, which was digested and cloned into pKNT25 similarly treated, leading to the construction of pIG20. The *frlM* ORF was amplified by PCR using primers ARA1033 and ARA1050, designed to introduce KpnI and XbaI restriction sites, digested with the same enzymes and cloned into pKT25, originating pIG23. All PCR amplifications described here were performed using chromosomal DNA of *B. subtilis* 168T^+^ as template. *E. coli* BTH101 was co-transformed with one pUT18 and one pKT25/pKNT25 derivatives, and the resulting co-transformants were selected in LA supplemented with ampicillin (100 μg mL^-1^), kanamycin (30 μg mL^-1^) and streptomycin (100 μg mL^-1^). These co-transformants were inoculated in LB with the respective antibiotics and IPTG (0.5 mM), grown aerobically at 30ºC and protein-protein interaction was measured by β-galactosidase activity following Euromedex instructions, which was expressed in Units mg^-1^ dry weight bacteria.

# Supplementary references

1. Arnaud M, Chastanet A, Débarbouillé M. 2004. New vector for efficient allelic replacement in naturally nontransformable, low-GC-content, gram-positive bacteria. Appl Environ Microbiol 70:6887–6891. <https://doi.org/10.1128/AEM.70.11.6887-6891.2004>

2. Perego, M. 1993. Integrational vectors for genetic manipulation in *Bacillus subtilis*. p. 615–624. In A. L. Sonenshein, J. A. Hoch, and R. Losick (ed.), *Bacillus subtilis* and other gram-positive bacteria: biochemistry, physiology, and molecular genetics. American Society for Microbiology, Washington, DC. <https://doi.org/10.1128/9781555818388.ch42>

3. Ferreira MJ, de Sá-Nogueira I. 2010. A multitask ATPase serving different ABC-type sugar importers in *Bacillus subtilis*. J Bacteriol 192:5312–5318. <https://doi.org/10.1128/JB.00832-10>

4. Anagnostopoulos C, Spizizen J. 1961. Requirements for transformation in *Bacillus subtilis*. J Bacteriol 81:741–746. <https://doi.org/10.1128/jb.81.5.741-746.1961>

5. Karimova G, Ullmann A, Ladant D. 2000. A bacterial two-hybrid system that exploits a cAMP signaling cascade in *Escherichia coli*. Methods Enzymol 328:59–73. <https://doi.org/10.1016/s0076-6879(00)28390-0>
